# Comparative Evaluation of Functional and Quality of Life Outcomes in Conventional and Soft-Liner Relined Obturators for Patients With Maxillofacial Defects

**DOI:** 10.7759/cureus.72405

**Published:** 2024-10-25

**Authors:** Anaida Acharya, Tapan K Patro, Angurbala Dhal, Lokanath Garhnayak, Ullash Kumar

**Affiliations:** 1 Prosthodontics, Sriram Chandra Bhanja (SCB) Dental College and Hospital, Cuttack, IND

**Keywords:** masticatory performance, maxilla-facial prosthodontics, maxillectomy rehabilitation, maxillofacial obturator, praat, silicone soft liner

## Abstract

Background and objective

Oral cancer is the sixth most common cancer worldwide. The prevalence of mucormycosis, a progressive fungal infection affecting the nasal and paranasal sinuses, is also on the rise, especially in patients after COVID-19 treatment. Surgical resection frequently employed in these cases results in maxillary defects which lead to functional and aesthetic impairments. Obturator prostheses are used for the rehabilitation of these maxillofacial defects. However, the choice of material and design can have an impact on patient outcomes. The present study aims to compare the maxillofacial obturators lined with a soft-liner, in terms of masticatory performance, speech parameters, and quality of life (QoL) of patients with maxillofacial defects with conventionally used obturators.

Materials and methods

This in vivo study was conducted in P.G. Department of Prosthodontics and Crown & Bridge, S.C.B. Dental College and Hospital, Cuttack after receiving approval from the Institutional Ethical Committee and Clinical Trial Registry-India (CTRI) (Reg. No. Trial REF/2022/07/056836). In this cross-over trial, 27 patients meeting the inclusion and exclusion criteria were provided with a conventional maxillofacial obturator which was later on relined with a tissue soft-liner. Parameters such as masticatory performance, speech and QoL were evaluated for both conventional and relined prosthesis. The masticatory performance was evaluated using a colour-changing chewing gum with a scoring system based on the colour change observed after 60 seconds of chewing the gum.

Results

The masticatory performance was better with the relined obturator (mean: 3.22) compared to the conventional obturator (2.74) yielding a statistically significant result (p=0.028). Analysis of speech parameters showed that the relined dentures had lower jitter (2.13 vs. 2.47) with a statistically significant result (p=0.012). Parameters such as shimmer and fundamental frequency, although not statistically significant, were lower for the relined prosthesis compared to their conventional counterparts. Also, the participants with soft-liner relined obturators had higher quality of life (mean: 170.4) than participants with conventional obturators (mean: 179.1). However, this difference was not statistically significant (p=0.42).

Conclusions

The present study demonstrates that soft-liner relined obturators offer superior masticatory performance and speech outcomes compared to conventional obturators in patients with maxillofacial defects. These findings suggest that the use of soft-liner relined obturators may be a more effective option for rehabilitating patients with maxillofacial defects, improving their functional and aesthetic outcomes. Although quality of life scores were higher for participants with soft-liner relined obturators, the difference was not statistically significant. Further studies with larger sample sizes and longer follow-up periods are recommended to confirm these results.

## Introduction

Oral cancer is a global concern and ranks as the sixth most common cancer worldwide [1]. Apart from this, mucormycosis has also emerged as a potentially fatal fungal infection that has been a pressing global issue post-COVID. According to estimates, the prevalence of mucormycosis in India is 140 cases per million people, around 80 times greater than in any affluent nation [2]. Treatment of oral cancer and rhinocerebral mucormycosis often needs surgical resection of the maxilla and surrounding structures, resulting in communication between the oral and nasal cavities [3]. Apart from the resulting oro-nasal community, there is also loss of cheek and lip support, aesthetic defects of the middle third of the face, and defects in speech and swallowing which can be managed by either reconstructive surgery or rehabilitated using a maxillofacial obturator prosthesis. Even though reconstructive surgery improves patient comfort, it is contraindicated when the defect is substantially large or when radiation therapy is being used in conjunction with the treatment [4].

Polymethyl methacrylate (PMMA) which is commonly employed for the fabrication of obturator prosthesis has a significant shortcoming; owing to its rigidity, it is difficult to incorporate all the anatomical undercuts in the prosthesis which shall help in its retention [5]. Relining the intaglio surface of the obturator in such cases with a flexible soft lining material enhances patient comfort because of improved retention and provides a cushioning effect that evenly distributes the stresses over the lining mucosal surface [6]. The success of an obturator prosthesis can be defined when several checkboxes including masticatory efficiency, speech, swallowing, and aesthetics are checked, all of which ultimately result in improved patient Quality of Life (QoL). Thus, in the present study, we aimed to evaluate whether relining an obturator with a soft-lining material resulted in an improvement of such parameters or not.

## Materials and methods

This crossover clinical trial was conducted in P.G. Department of Prosthodontics and Crown & Bridge, S.C.B. Dental College and Hospital, Cuttack after receiving approval from the Institutional Ethical Committee and Clinical Trial Registry-India (CTRI) (Reg. No. Trial REF/2022/07/056836). In this study, 27 patients meeting the inclusion and exclusion criteria were provided with a conventional maxillofacial obturator which was later on relined with a tissue soft-liner. A number of parameters, including speech, QoL, and masticatory performance, were assessed for both conventional and relined prostheses.

Inclusion criteria

Patients, who required an obturator prosthesis, were selected after primary healing from surgical trauma or acute infection control, had at least two functional occlusal units, maxillary defects classified under Aramany class I to VI defects or/and soft palate defects, and age group of 18 years and above.

Exclusion criteria

Patients who had physical, psychiatric and neurological disability that interfered with the study, had speech impairment not related to maxillary defect, minimum jaw opening of 15mm or less, mouth opening restriction due to fibrosis, patients who had undergone free flap closure or free flap bone reconstruction, and had temporomandibular joint (TMJ) disorder.

Materials

**Figure 1:**
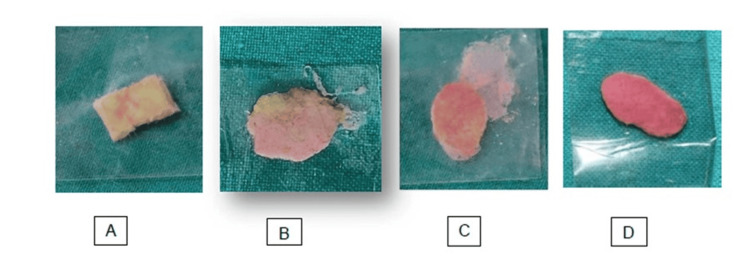
Colour changing chewing gum: A- Score 2, B- Score 3, C- Score 4, D- Score 5

The obturators were fabricated for each patient conventionally using heat cure acrylic material (DPI, Mumbai). Soft liner material (Ufi Gel P, VOCO, Germany) was used to reline the obturator at a later stage. A colour-changing chewing gum (Masticatory Performance Evaluating Gum XYLITOL; Lotte Co., Ltd., Tokyo, Japan) was used to assess the masticatory performance, and a grading system was established based on the colour shift that was seen after 60 seconds of chewing the gum. The colour of the chewed bolus was classified into five categories based on colour green, yellow, light pink, pink and red. The scoring was counted as i) green colour - 1 point, ii) yellow colour - 2 points, iii) light pink - 3 points, iv) pink - 4 points and v) red - 5 points (Figure 1). Score 5 indicated the best masticatory performance.

Speech parameters such as jitter, shimmer, and fundamental frequency during phonation of vowels /a/ /i/ & /u/ were evaluated using the PRAAT software (Institute of Phonetic Sciences, University of Amsterdam, 1995) [7]. Currently, acoustic parameters commonly used in applications of acoustic analysis as well as the most referenced in the literature, are the fundamental frequency (F0), jitter, shimmer, harmonic-to-noise ratio (HNR) and frequency formants. The measure of these parameters is performed in a recorded speech signal with the patient/control producing a long steady state vowel. Measurements of F0 disturbance, jitter and shimmer have proven to be useful in describing the vocal characteristics and thus these have been selected to study change in speech:

1. Fundamental frequency (F0) - the rate of vibration of the vocal folds. 2. Jitter - the measure of the cycle-to-cycle variations of the fundamental glottal period. 3. Shimmer - the cycle-to-cycle variation in amplitude.

Quality of life was assessed using the European Organization of Research and Treatment of Cancer Quality of Life Core Questionnaire (EORTC QLQC30) and Head and Neck-Specific Questionnaire (EORTC QLQ-H&N35) [3].

Methodology

**Figure 2:**
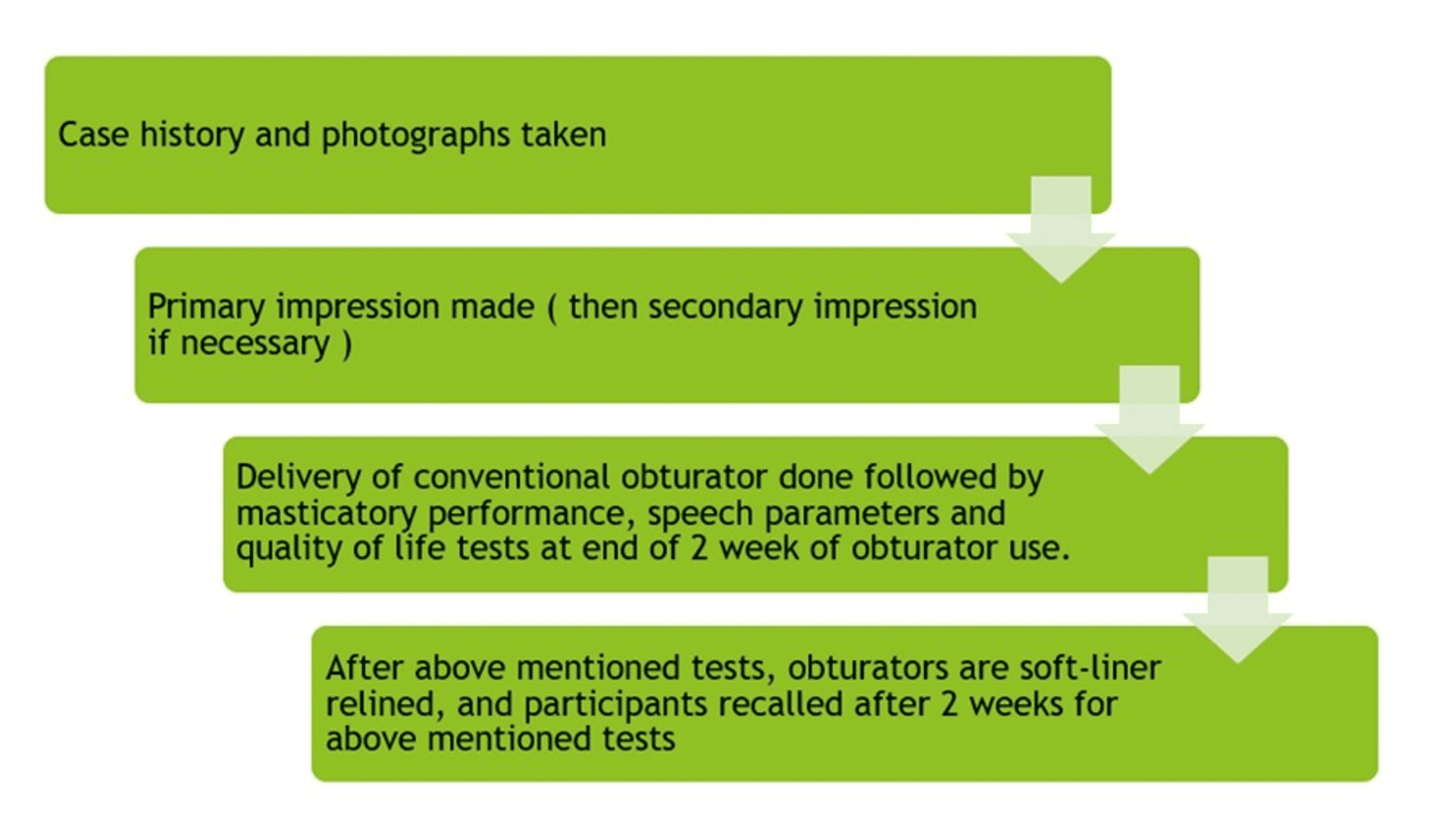
Study design flow chart

All the patients included in the study received a conventional maxillary obturator fabricated using heat cure acrylic resin after a healing of about three to four months post-operatively from the day of surgery. The parameters discussed above were evaluated at the end of two weeks and the denture was relined with a soft liner material. Further evaluation for the soft-lined denture was done after two weeks and the results were tabulated for analysis. The study design flow chart is depicted in Figure 2.

The masticatory efficacy evaluated using the novel colour-changing gum was given a score from 1 to 5 after the patient chewed it for 60 seconds. The chewed gum was mounted between two glass slides and pressed to a thickness of approximately 1mm, followed by the placement of a clear film with 2mm square marks. Each small square was given a point from 1 to 5 based on the colour change (green, yellow, light pink, pink, and red respectively) and the mean score was calculated. The acoustic analyses in this study were conducted using PRAAT software (Institute of Phonetic Sciences, University of Amsterdam) to evaluate jitter (how much a given period differs from the period that immediately follows it), shimmer (the cycle-to-cycle variation in amplitude) and fundamental frequency (rate of vibration of the vocal folds) between conventional and relined prosthesis. QoL assessment was done using the questionnaires described previously.

Parametric tests (unpaired-T test) were used for the evaluation of data, the distribution of which was normal and continuous, analysed using SPSS 12.0 for Windows (SPSS Co., Ltd., Chicago, USA). Statistical significance was set at p <0.05.

## Results

Twenty-seven patients included in the study had a mean age of 53.89 years, with a male: female ratio of 2.375:1 (n = 19:8). Based on medical history, 10 patients (37.03%) had mucormycosis while 17 patients (62.97%) had undergone surgical resection for oral squamous cell carcinoma (Table 1).

**Table 1:** Patient demography N denotes the number of patient.

Characteristic	N	Percentage
Age (in years) 20-30 yrs	2	7.4
31-40 yrs	1	3.7
41-50 yrs	4	14.8
51-60 yrs	11	40.7
>60 yrs	9	33.4
Male	19	72.4
Female	8	29.6
Past History Mucormycosis	10	37.03
Squamous cell carcinoma	17	62.97

**Figure 3:**
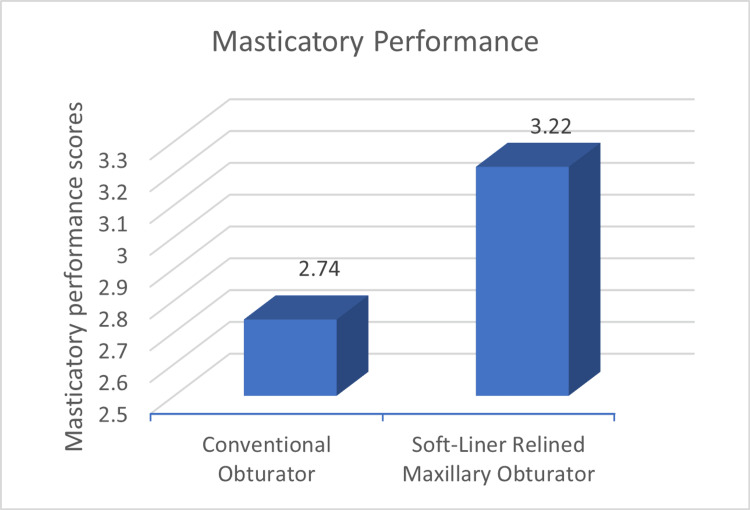
Mean masticatory performance between conventional and soft-liner relined obturator The numerical value represents the masticatory performance score.

The masticatory performance was better for the relined group as indicated by the mean score of 3.22 compared to 2.74 of the conventional group with a p-value of 0.028 yielding a statistically significant difference (Figure 3).

**Figure 4:**
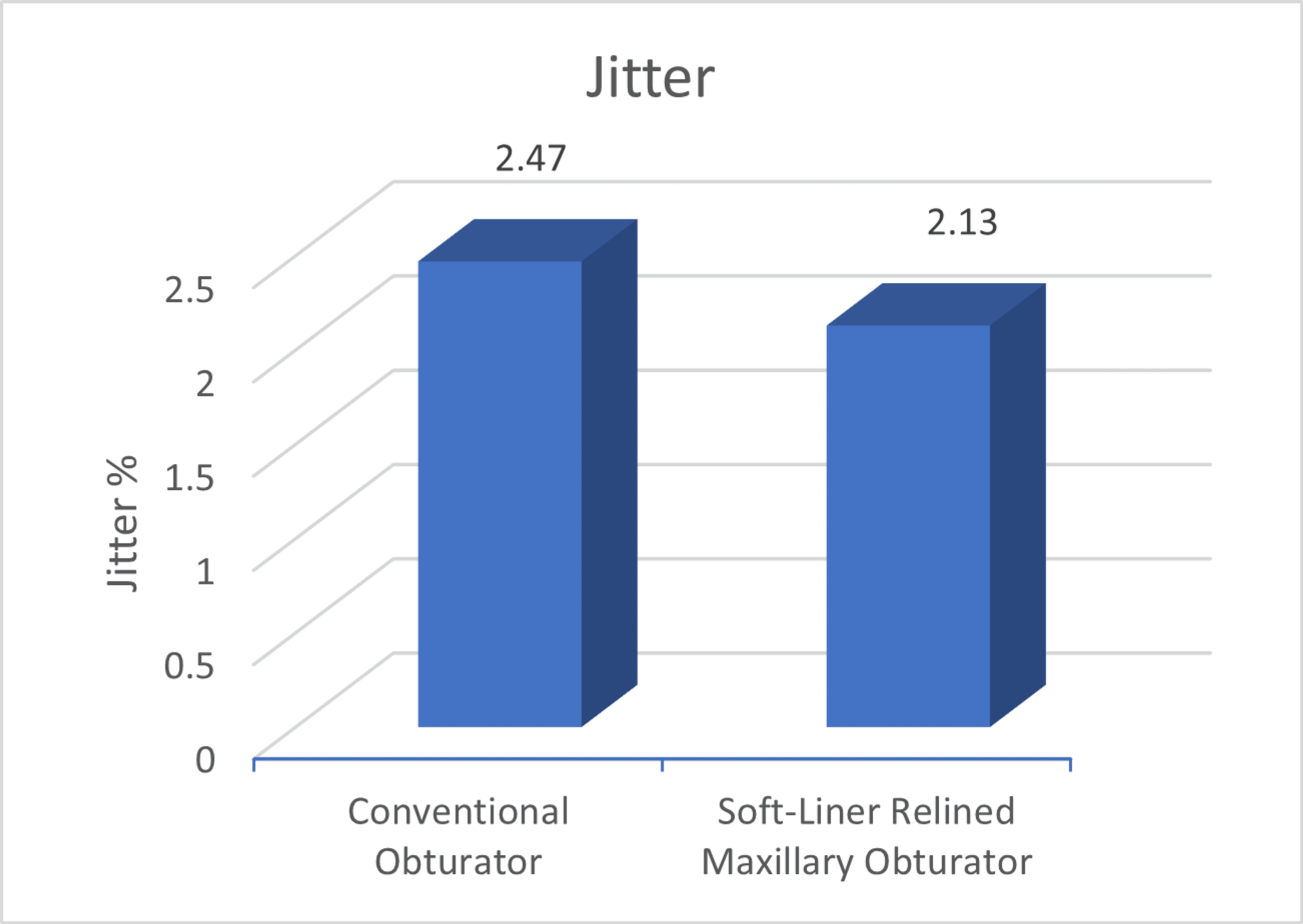
Mean Jitter values between conventional and soft-liner relined obturator The numerical value denotes Jitter % (Acoustic parameter to evaluate speech).

Speech analysis parameters showed that the jitter percentage was significantly lower for the relined group (2.13 Jitter% ) compared to conventional obturators (2.47 Jitter%) (Figure 4).

**Figure 5:**
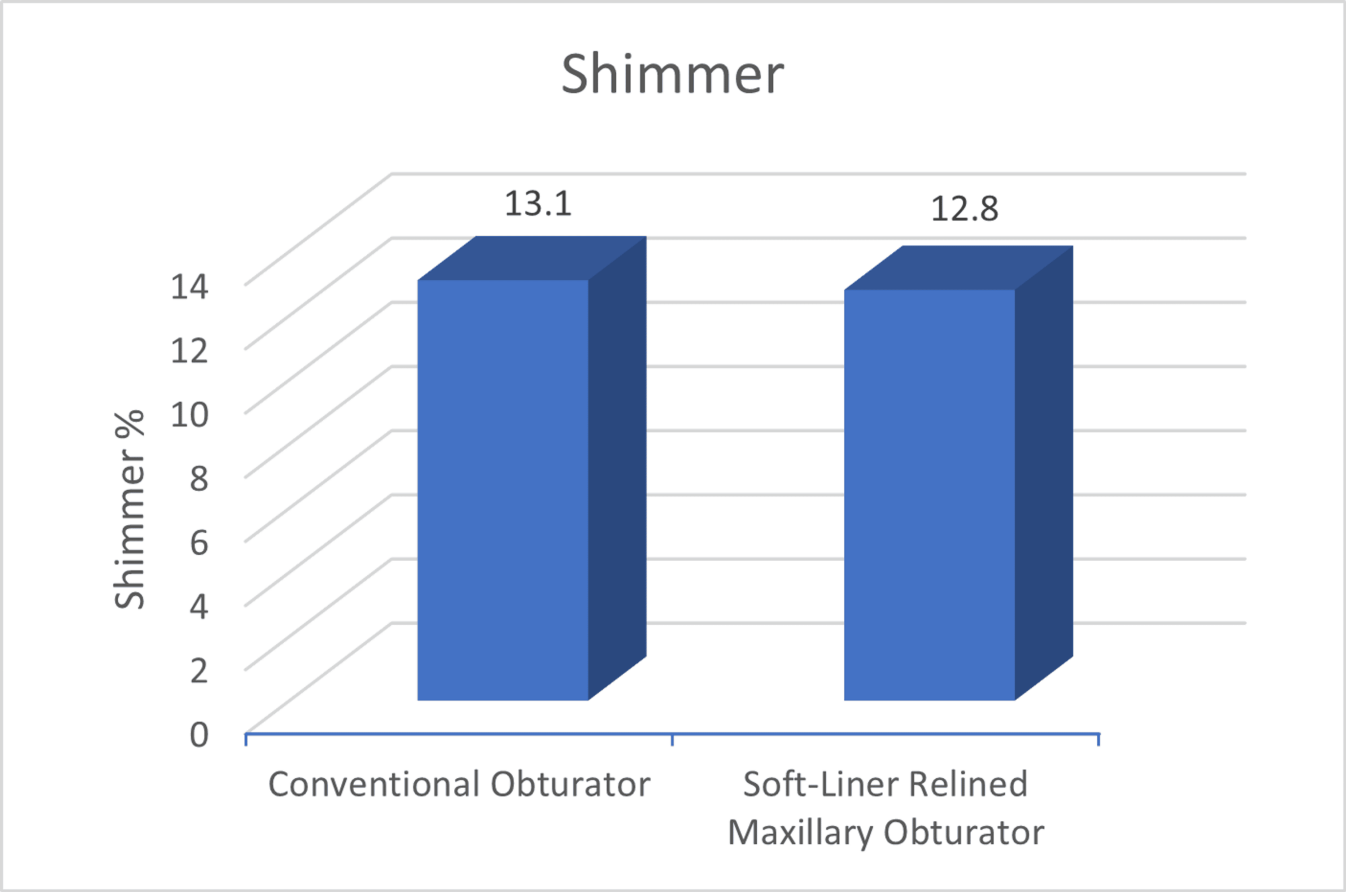
Mean Shimmer values between conventional and soft-liner relined obturator The numerical value denotes the shimmer % (acoustic parameter to evaluate speech).

**Figure 6:**
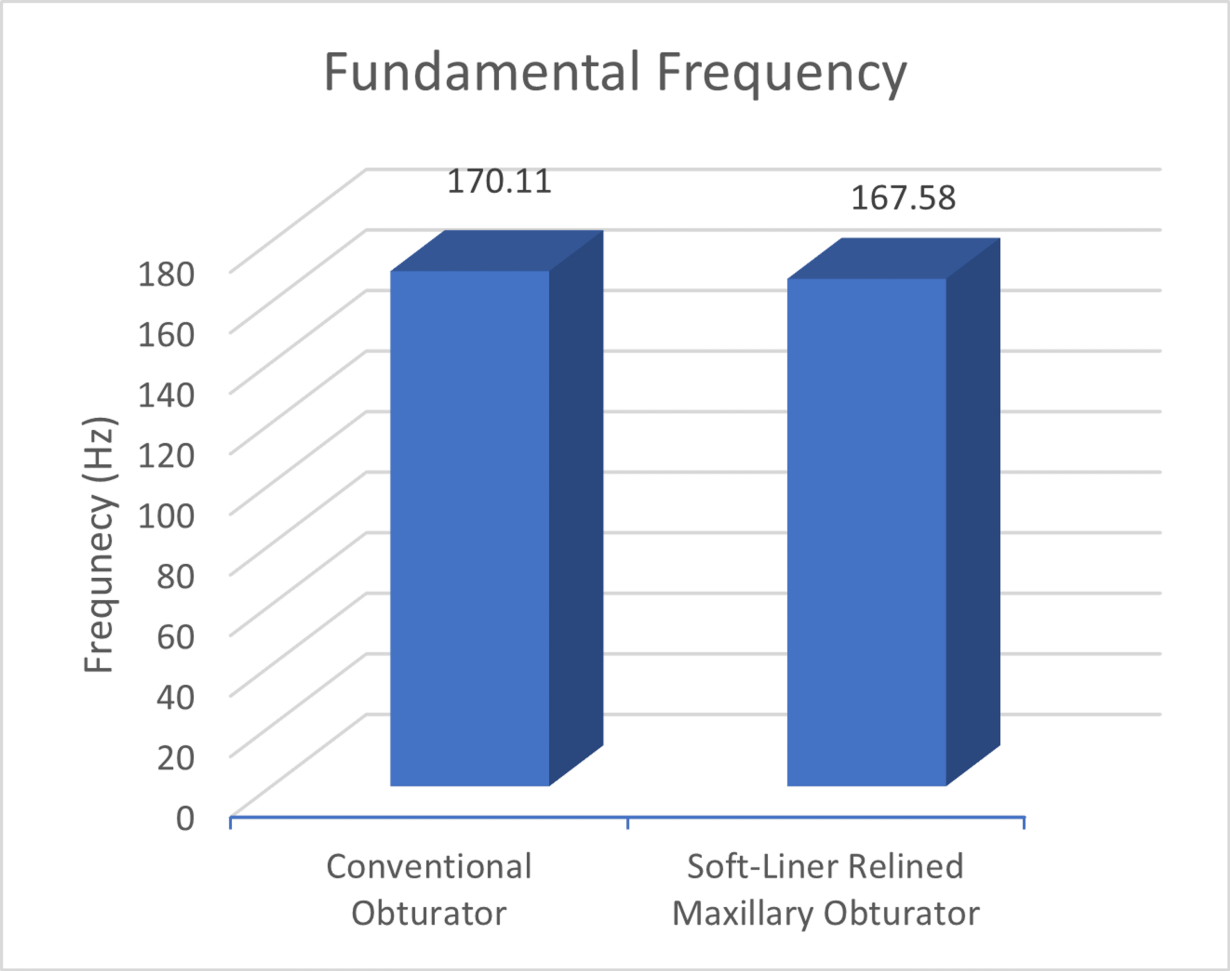
Mean fundamental frequency between conventional and soft-liner relined obturator

Other parameters such as shimmer and fundamental frequency were also better for the relined obturators (12.8 shimmer%; 167.58 Hz) compared to the conventional obturators (13.1 shimmer%; 170.11 Hz), the difference, however, was not statistically significant (Figures 5, 6).

**Figure 7:**
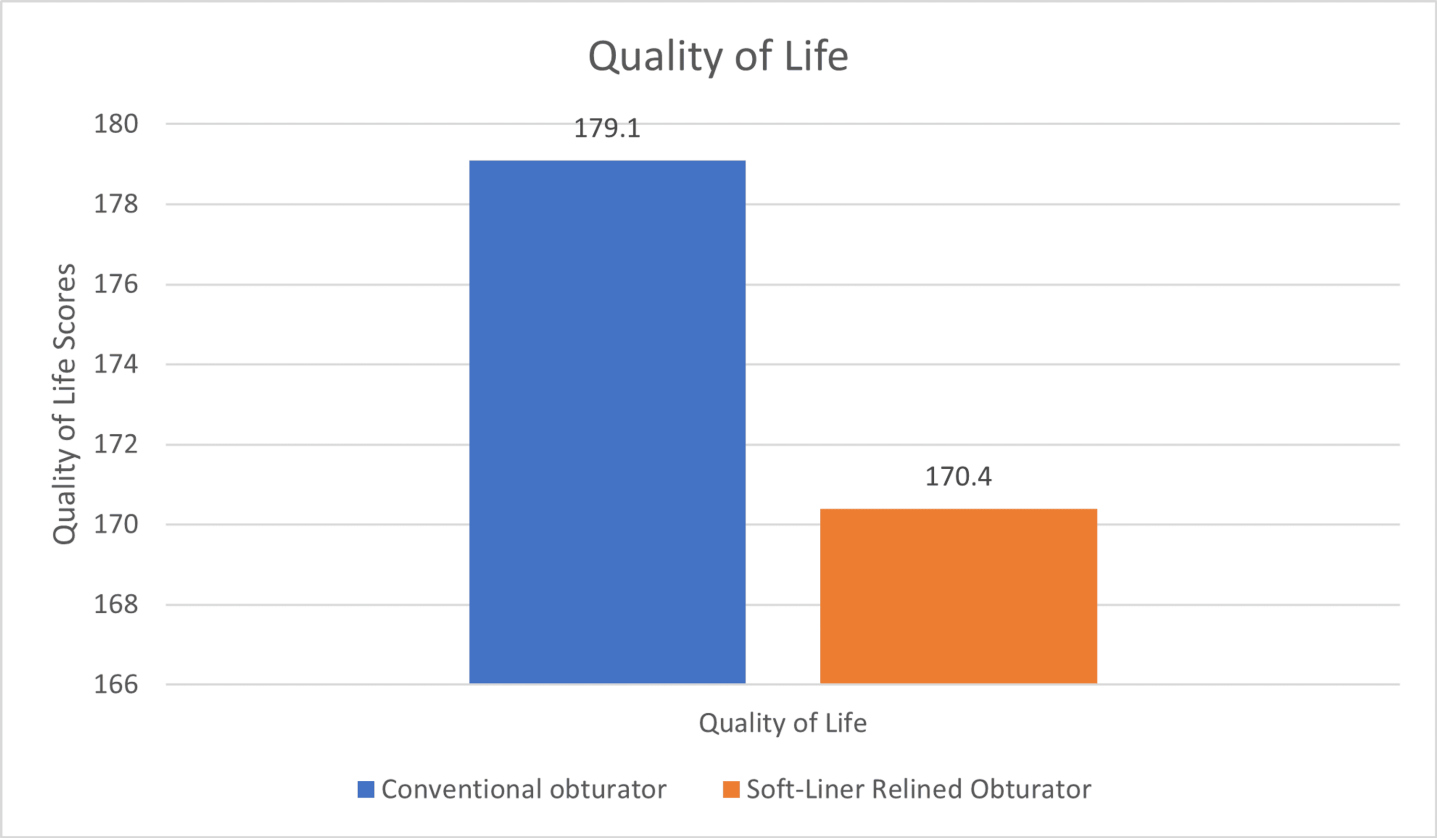
Mean Quality of Life values between conventional and soft-liner relined obturator

Analysis of Quality of Life revealed better outcomes for the soft liner group as indicated by the mean score of 170.4 which was better than the score 179.1 achieved by the conventional group (lower value implies better QoL), however, the difference was not statistically significant (Figure 7) (Table 2).

**Table 2:** Comparison of Conventional and Soft liner relined obturators using different parameters N- Number, SD- Standard deviation, statistically significant at *p<0.05 using unpaired t-test.

Parameter	Conventional obturator (n=27)		Soft liner relined obturator (n=27)		
	Mean	SD	Mean	SD	P-value
Masticatory performance score	2.74	0.71	3.22	0.84	0.028*
Jitter (Jitter %)	2.47	0.54	2.13	0.43	0.012*
Shimmer (Shimmer %)	13.1	2.65	12.8	2.3	0.7
Fundamental frequency (Hz)	170.11	17.4	167.58	11.65	0.53
Quality of Life Score	179.1	39.5	170.4	39.2	0.42

## Discussion

Surgical resection of the maxilla in patients suffering from oral cancer and/or mucormycosis is often indicated which results in a maxillofacial defect. Most of the cases cannot be corrected by means of surgical closure. Maxillofacial obturator prosthesis is a boon for such patients by managing the functional and aesthetic outcomes and improving the quality of life of such patients [5,8-10]. The conventional obturators fabricated from heat cure acrylic resin material are often limited due to their unyielding nature as they are unable to engage the anatomic undercuts without damaging the oral tissues [5]. The soft liner silicone material is often used as a relining material for overcoming this issue [6], the outcomes of which have been analyzed in the present study comparing it to the conventionally fabricated prosthesis. Silicone relining on PMMA prosthesis resulted in benefits such as seal improvement, enhanced retention at the defect site and improved phonetics. Silicone also had its own disadvantage of increased microbial adhesion but with sanitary care and regular follow-up, this could be overcome. The bonding between silicone and acrylic had also been under scrutiny in the past but with advancing science in dental materials, it is now possible to have an effective bond between them with the help of adhesive.

Comparison of masticatory performance, speech parameters as well as quality of life between the conventional and soft liner relined materials revealed differences between the two groups. The statistically significant differences between the two groups in terms of masticatory outcomes are in alignment with previous researches. The use of colour-changing chewing gum for such evaluation has been well documented in the literature [11-14]. Yanamoto et al. [15] have compared the silicone-lined obturators with conventional obturators for masticatory efficacy using a chewing gum. The gum was used to collect saliva after 20 seconds and glucose extraction was done. The results for the relined prosthesis (median IQR 126.0 mg/dl) were significantly improved compared to the conventional group (median IQR 99.6 mg/dl). In the present study, masticatory performance which was assessed using the colour-changing chewing gum revealed a statistically significant improvement in soft-liner relined obturators when compared to conventional PMMA obturators, as the mean masticatory score in relined obturators was higher than conventional.

Alteration of speech is a frequent finding in post-maxillectomy cases [3]. It has been documented that acoustic parameters like fundamental frequency (F0), jitter, shimmer, harmonic-to-noise ratio (HNR), and frequency formants which are commonly used in acoustic analysis help in the description of vocal characteristics [7]. The present study evaluated jitter, shimmer and F0 between the two groups and found better outcomes for these parameters with the relined obturators with statistically significant differences reported for jitter. Rieger et al. [16] have reported that maxillofacial obturator can significantly help in restoring the speech to pre-operative levels. Possibly the speech outcomes can be further improved by customising the obturator’s palatal contours by palatogram as reported in the study conducted by Grover et al. [17]. The normal value of jitter in adult individuals ranges from 0%-1%. In this study, we observed the jitter values reduced with the use of a soft-liner relined obturator when compared to the jitter values while using conventional obturators which signifies that soft-liner relining obturator leads to jitter values closer to the jitter value of healthy individuals. This trend was similar for acoustic parameters of shimmer and fundamental frequency.

The questionnaires most commonly used to assess the quality of life in head and neck cancer patients were EORTC QLQ-C30 and EORTC QLQ-H&N35 which have been translated and validated in several languages [3]. They have also been translated and validated in Hindi and other Indian vernacular languages by Chaukar et al. [18]. Evaluation of the patients with these questionnaires for Quality of Life using conventional and relined prostheses revealed a better outcome for the latter group, although the difference was not statistically significant. The findings of the present study are in agreement with that of Yanamoto et al. [15] who utilised the oral health-related Quality of Life Questionnaire for evaluation and found better results for the silicone-relined group.

Further studies with a larger sample size can be formulated to evaluate the findings of the present study. Limitations in the present study were that the extent of natural dentition, which can significantly affect the masticatory performance and act as a confounding factor, was not considered, and the hygiene maintenance of the obturators was not taken into consideration. The present study has also not delineated patients on the basis of the extent of defect, another confounding variable which can have an influence on the parameters assessed. Future studies can study the hygiene maintenance of silicone-relined obturators.

## Conclusions

This study reveals that soft-liner relined obturators significantly enhance masticatory function in individuals with maxillofacial defects, surpassing the effectiveness of conventional obturators. The findings imply that soft-liner relined obturators enhance the speech parameters of the patient and may be a superior choice for rehabilitation, yielding improved functional and aesthetic results. While the quality of life scores showed a positive trend with soft-liner relined obturators, further research with expanded sample sizes and extended follow-up periods is necessary to substantiate these outcomes and fully explore the benefits of this treatment approach.
